# Supplementary material for: Expression and characterization of thermotolerant lipase with broad pH profiles isolated from an Antarctic Pseudomonas sp strain AMS3
Source: PeerJ. 2016 Oct 20;4:e2420. doi: 10.7717/peerj.2420 (PMC5075702; doi:10.7717/peerj.2420)
Supplement: Data S1 [file peerj-04-2420-s001.docx]

| Colony | Tributyrin  agar | Rhodamine agar | Victoria blue agar |
| --- | --- | --- | --- |
| 1 | + | + | + |
| 2 | + | + | + |
| 3 | + | - | - |

Table 1: Qualitative determination of recombinant *E.coli* harbouring putative lipase gene. Major lipid component in the Rhodamine B and Victoria blue agar plates contain olive oil and triolein, respectively

Purification table:


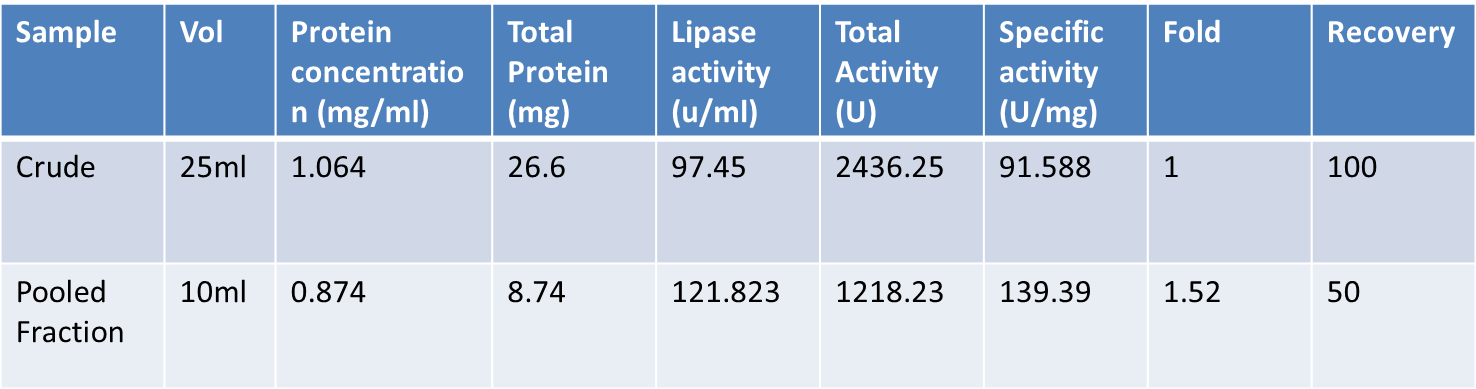


Effect of temperature on lipase activity

| Temperature | U/mg | Error (+-) |
| --- | --- | --- |
| 5 °C | 6.22 | 0.093 |
| 10 °C | 8.29 | 0.082 |
| 20 °C | 17.73 | 0.16 |
| 30 °C | 34.48 | 0.52 |
| 40 °C | 66.20 | 1.3 |
| 50 °C | 75.41 | 1.5 |
| 60 °C | 70.81 | 1.34 |
| 70 °C | 11.98 | 0.131 |

Effect of temperature on stability

| Temperature | Relative activity % |
| --- | --- |
| 5 °C | 88 +-0.2 |
| 10 °C | 85 +-0.5 |
| 20 °C | 91 +-0.9 |
| 30 °C | 100 +-0.95 |
| 40 °C | 98 +-1 |
| 50 °C | 85 +-1.1 |
| 60 °C | 65 +-0.2 |
| 70 °C | 18 +-0.2 |

Effect of pH on activity.

| pH | U/mg | Error (+-) |
| --- | --- | --- |
| SA 4 | 2.88 | 0.026 |
| SA 5 | 41.37 | 0.39 |
| SA 6 | 39.93 | 1.1 |
| PB 6 | 44.50 | 2.45 |
| PB 7 | 48.20 | 2.5 |
| PB 8 | 50.37 | 2.53 |
| TH 8 | 49.08 | 2.44 |
| TH 9 | 32.73 | 1.3 |
| GN 9 | 32.38 | 1.46 |
| GN10 | 24.36 | 0.51 |
| GN11 | 11.14 | 0.12 |
| GN12 | 3.95 | 0.03 |

Effect of pH stability

| pH | Relative activity % |
| --- | --- |
| SA 4 | 2.66+-2.1 |
| SA 5 | 72.11+-1.7 |
| SA 6 | 83.32+-3.2 |
| PB 6 | 89.45+-4 |
| PB 7 | 100+-4 |
| PB 8 | 95.01+-3.7 |
| TH 8 | 95.69+-2.9 |
| TH 9 | 85.03+-3.9 |
| GN 9 | 94.48+-2.1 |
| GN10 | 56.3+-2.3 |
| GN11 | 12.4+-2.1 |
| GN12 | 4.98+-1.7 |

Effect of metal ion

|  | 1 mM | Error(+-) | 5 mM | Error(+-) |
| --- | --- | --- | --- | --- |
| Control | 100 | 2.9 | 100 | 2.5 |
| Li^+^ | 161 | 3.2 | 28.3 | 0.75 |
| Na^+^ | 104.6 | 3.0 | 98 | 3.8 |
| K^+^ | 83.4 | 2.1 | 122.4 | 4.1 |
| Rb^+^ | 161.9 | 3.0 | 83.6 | 3.2 |
| Mg^2+^ | 89.1 | 2.9 | 61.2 | 2.9 |
| Ca^2+^ | 72.3 | 2.1 | 55.7 | 3.7 |
| Mn^2+^ | 70.5 | 0.9 | 55.5 | 2.5 |
| Fe^2+^ | 88.7 | 1.2 | 44.4 | 2.4 |
| Co^2+^ | 88.9 | 1.7 | 100 | 4 |
| Ni^2+^ | 61 | 0.9 | 33.4 | 0.9 |
| Zn^2+^ | 150 | 3.2 | 72.3 | 1.2 |

Effect of Organic solvent:

| Organic solvent | Relative activity % |
| --- | --- |
| Control | 100+-2.1 |
| DMSO | 73.78+-3.7 |
| Methanol (-0.76) | 88.91+-3.1 |
| Acetonitril (-0.33) | 7.96+-4.5 |
| Ethanol (-0.24) | 36.22+-4.1 |
| Acetone (-0.24) | 25.9+-3.9 |
| 1-propanol (0.28) | 6.58+-4.8 |
| Chloroform (2.0) | 20.74+-6.1 |
| Benzene (2.0) | 42.12+-5.5 |
| Toulene (2.5) | 30.22+-5.2 |
| Xylene (3.1) | 40.17+-3.9 |
| n-hexane (3.5) | 116.58+-4.7 |
